# Macrophage migration inhibitory factor promotes renal injury induced by ischemic reperfusion

**DOI:** 10.1111/jcmm.14234

**Published:** 2019-04-09

**Authors:** Jin H Li, Ying Tang, Jun Lv, Xiao H. Wang, Hui Yang, Patrick M. K. Tang, Xiao R. Huang, Zhi J. He, Zi J Zhou, Qiu Y. Huang, Jörg Klug, Andreas Meinhardt, Günter Fingerle‐Rowson, An P. Xu, Zhi H. Zheng, Hui Yao Lan

**Affiliations:** ^1^ Department of Nephrology The Seventh Affiliated Hospital of Sun Yat‐sen University Sun Yat‐sen University Shenzhen China; ^2^ Department of Medicine and Therapeutics Department of Anatomical and Cellular Pathology Li Ka Shing Institute of Health Sciences the Chinese University of Hong Kong Hong Kong China; ^3^ Department of Nephrology Sun Yat‐sen Memorial Hospital Sun Yat‐Sen University Guangzhou China; ^4^ Department of Anatomy and Cell Biology Justus‐Liebig‐University Giessen Germany; ^5^ Department I of Internal Medicine University Hospital Cologne, and Center for Integrated Oncology Köln‐Bonn Cologne Germany

**Keywords:** acute kidney injury, cytokines, macrophage migration inhibitory factor (MIF), renal inflammation

## Abstract

Macrophage migration inhibitory factor (MIF) is pleiotropic cytokine that has multiple effects in many inflammatory and immune diseases. This study reveals a potential role of MIF in acute kidney injury (AKI) in patients and in kidney ischemic reperfusion injury (IRI) mouse model in MIF wild‐type (WT) and MIF knockout (KO) mice. Clinically, plasma and urinary MIF levels were largely elevated at the onset of AKI, declined to normal levels when AKI was resolved and correlated tightly with serum creatinine independent of disease causes. Experimentally, MIF levels in plasma and urine were rapidly elevated after IRI‐AKI and associated with the elevation of serum creatinine and the severity of tubular necrosis, which were suppressed in MIF KO mice. It was possible that MIF may mediate AKI via CD74/TLR4‐NF‐κB signalling as mice lacking MIF were protected from AKI by largely suppressing CD74/TLR‐4‐NF‐κB associated renal inflammation, including the expression of MCP‐1, TNF‐α, IL‐1β, IL‐6, iNOS, CXCL15(IL‐8 in human) and infiltration of macrophages, neutrophil, and T cells. In conclusion, our study suggests that MIF may be pathogenic in AKI and levels of plasma and urinary MIF may correlate with the progression and regression of AKI.

## 1 INTRODUCTION

Macrophage migration inhibitory factor (MIF) is a pleiotropic protein and has multiple roles in both innate and adaptive immunity.^1^, ^2^ Many studies have shown that MIF is protective in many inflammatory and immune diseases including sepsis, rheumatoid arthritis and systemic lupus erythematosus.^3^, ^4^, ^5^ MIF also participates in many inflammatory kidney diseases,^6^ including rapidly progressive glomerulonephritis,^7^ lupus nephritis,^8^ anti‐glomerular basement membranous (GBM) glomerulonephritis,^9^ anti‐neutrophil cytoplasmic antibodies (ANCA)‐associated vasculitis,^10^ renal allograft rejection^11^, ^12^ and acute kidney injury (AKI).^13^ Clinically, plasma MIF levels are significantly elevated in patients with septic shock and MIF is a potential biomarker for renal replacement therapy for AKI after liver transplantation.^14^, ^15^ In addition, increased renal MIF levels are also reported in a mouse model of IRI‐AKI.^16^ All these observations suggest that MIF may be important in AKI. However, the role and mechanisms of MIF in AKI remains to be further investigated.

In the present study, we first demonstrate that both serum and urinary levels of MIF are highly elevated in AKI patients and associated with the onset, the severity and the recovery of AKI. We also uncover the pathogenic role of MIF in AKI induced in MIF knockout (KO) mice by using an IRI‐AKI mouse model, a common cause of AKI. Furthermore, the potential role and mechanisms of MIF in the pathogenesis of AKI are investigated.

## 2 METHODS

### 2.1 Patients with AKI

According to RIFLE criteria (Figure S1A), a total of 47 patients with AKI and 30 healthy controls without evidence for any abnormalities or diseases from the Physical Examination Center were included in this study. AKI patients with diabetes, chronic kidney disease (CKD) and multiorgan dysfunction syndrome (MODS) were excluded from the study. Of 47 AKI patients, 10 patients were sequentially measured for both plasma and urinary MIF levels over a period of 14 days from initial onset of AKI to the recovery phase, while other 37 patients received plasma and urinary MIF analysis at the onset of AKI. The onset of AKI was defined at the first day of AKI, while the recovery stage of AKI was defined when serum creatinine returned to the baseline level. Serum creatinine and urine output were measured every 2 days. All patients gave written and informed consent, and the study protocol was approved by the Institutional Review Board of Sun Yat‐sen Memorial Hospital.

Plasma and urinary levels of MIF were measured by using the Quantikine^®^ human MIF ELISA kit (R&D Systems), whereas serum creatinine was analysed using an automatic biochemical analyzer (Hitachi 7600, Tokyo, Japan).

### 2.2 Ischemic AKI mouse model

Male MIF KO mice and littermate WT mice (C57/BL6 background) at 8‐12 weeks of age (20‐25 g body weight) were used as described previously.^17^ To induce AKI, bilateral kidney artery clamping was applied for 45 min as described in our previous study.^18^ Mice were sacrificed at day 1 to day 3 after AKI operation. The operation protocols were approved by Animal Experimental Committee of The Chinese University of Hong Kong.

### 2.3 Renal function and histology

Serum was collected for renal creatinine detection from day 1 to day 3 after AKI. Serum creatinine was detected by using a direct Creatinine LiquiColor kit (Stanbio Laboratory, Boerne, TX, USA). Renal pathology is examined by periodate‐Schiff (PAS) staining (Sigma‐Aldrich, 395B, St. Louis, MO, USA), formalin‐fixed kidney tissues were embedded into paraffin and sectioned (3 μm), and then followed by PAS staining. The number of tubular necrosis was scored at ×40 magnification and a total of 10 fields of cortical versions were examined and calculated as previously study described.^19^, ^20^, ^21^

### 2.4 Immunohistochemistry

Immunohistochemistry was performed on paraffin embedded slides for renal MIF, CD74 expression, pro‐inflammatory cytokines as MCP‐1 and TNF‐α expression, and for infiltration of neutrophils, F4/80^+^ macrophages and CD3^+^ T cells as described previously.^9^, ^11^, ^22^, ^23^, ^24^ The catalogue number of the antibodies for immunohistochemistry as following: rabbit anti‐MIF (Santa Cruz, sc‐20121), goat anti‐CD74 (Santa Cruz, sc‐5438), goat anti‐MCP‐1 (Santa Cruz, sc‐1785), goat anti‐TNF‐α (Santa Cruz, sc‐1351), rabbit anti‐neutrophil (Santa Cruz, sc‐59338), rat anti‐F4/80 (Serotec, MCA497) and rabbit anti‐CD3 (Abcam, ab‐16669).

### 2.5 Western blot analysis

Western blotting was performed as described previously.^19^, ^20^, ^21^ We used the proteins from the renal cortex for Western blotting analysis. The catalogue number of antibodies used for Western blot were as following: rabbit anti‐MIF (Santa Cruz sc‐20121), goat anti‐CD74 (Santa Cruz, sc‐5438), rabbit anti‐phosphorylated NF‐κB p65 (Cell Signaling, No. 3031), mouse anti‐NF‐κB p65 (Cell Signaling, No. 6965), rabbit anti‐phosphorylated IkBα (Cell Signaling, 2859), anti‐IKBα (Cell Signaling, 9242L), rabbit anti‐TLR4 (Santa Cruz, sc‐10741) and mouse anti‐β‐actin (Santa Cruz, sc‐69879).

### 2.6 RT‐PCR analysis

mRNA expression levels were quantified with SYBR Green (Life Technologies, Carlsbad, CA). renal MIF, CD74, TNF‐α, MCP‐1, IL‐1β, IL‐6, CXCL15 (IL‐8), iNOS and GAPDH were measured with primers as previously described.^19^, ^20^, ^21^ The expressing levels of targeted genes were determined by normalizing to the housekeeping gene GAPDH. The primers of this cytokines were shown in Table S1.

### 2.7 Statistical analysis

Experimental and clinical data were statistically analysed using SPSS 19.0. GraphPad Prism (Prism 5.0; GraphPad software, La Jolla, CA, USA) was used to generate figures. All metric data were tested for normal distribution using the Shapiro‐Wilk W test. For clinical data, normally distributed data were compared using the Student's *t* test. Non‐normally distributed data were compared using the Mann‐Whitney *U* test. Non‐parametric data were compared using the Fisher's exact test. For correlation analysis, a linear regression analysis was performed using the Pearson correlation coefficient. For animal experimental data, results were presented as means ± SEM. The differences between groups were analysed by Student's *t* test. In all cases, *P *<* *0.05 was considered statistically significant, and two‐sided testing was used.

## 3 RESULTS

### 3.1 Serum and urinary MIF levels are significantly higher in patients with AKI and correlate with the progression or regression of AKI

**Figure 1:**
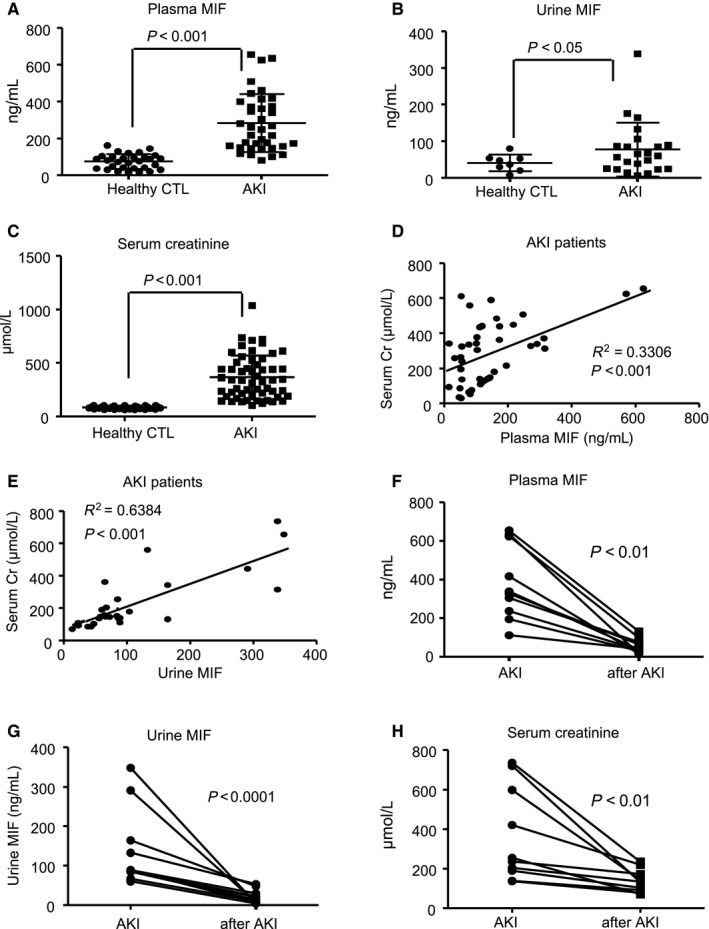
Plasma MIF and urinary MIF levels are elevated in patients with AKI and correlate closely with serum levels of creatinine. A and B, Plasma MIF and urinary MIF levels in healthy controls (n = 30) and AKI patients (n = 47) determined by ELISA. C, Serum creatinine levels in healthy controls and AKI patients. D and E, Linear correlation between plasma MIF or urinary MIF and serum creatinine in patients with AKI. F and G, Paired data of plasma MIF or urinary MIF levels at the onset and the recovery phase of AKI. H, Paired data of serum creatinine levels at the onset and the recovery phase of AKI. Each dot represents one normal or AKI patient

The baseline characteristics, comorbidities, causes of patients and healthy controls in this study were described in Tables 1 and 2, Figures S1B and S2. There were no differences in all above parameters between the patients and healthy control except the age. Plasma MIF levels were elevated in AKI patients compared to healthy controls (Table 2, Figure 1A). As shown in Table 2 and Figure 1B, elevated urinary MIF was also detected in these AKI patients. In addition, correlation analysis demonstrated that increased plasma and urinary MIF was closely correlated with elevated serum creatinine levels in patients with AKI (Figure 1C‐E). Interestingly, plasma MIF and urinary MIF levels largely declined in patients when AKI was resolved (Figure 1F‐H). These findings indicated that plasma MIF and urinary MIF levels were closely correlated with the progression or regression of AKI clinically.

**Table 1:** Causes of AKI

Parameter	Ischemic	Obstruction	Kidney diseases	Drugs	Sepsis	Allergy
Number	9	1	18	9	9	1
Gender (n)						
Male	9	0	9	7	7	1
Female	0	1	9	2	2	0
Age (years)	54 ± 22	42	47 ± 18	49 ± 26	68 ± 14	42

**Table 2:** Clinical information for 47 AKI patients and 30 healthy controls

	Healthy controls (n = 30)	AKI patients (n = 47)	*P*
Gender (n)			
Male	19	33	>0.05
Female	11	14	
Age (years)	30 ± 6	54 ± 20	<0.05
Serum Cr (μmol/L)	87.1 ± 14.54	366.48 ± 203.05	<0.01
Serum BUN (μmol/L)	4.49 ± 1.39	23.24 ± 16.13	<0.01
Plasma MIF (ng/mL)	74.35 ± 40.84	282.92 ± 166.63	<0.01
Urine MIF (ng/mL)	40.78 ± 22.52	75.47 ± 49.98	<0.05

### 3.2 MIF expression in a mouse model of ischemic AKI

**Figure 2:**
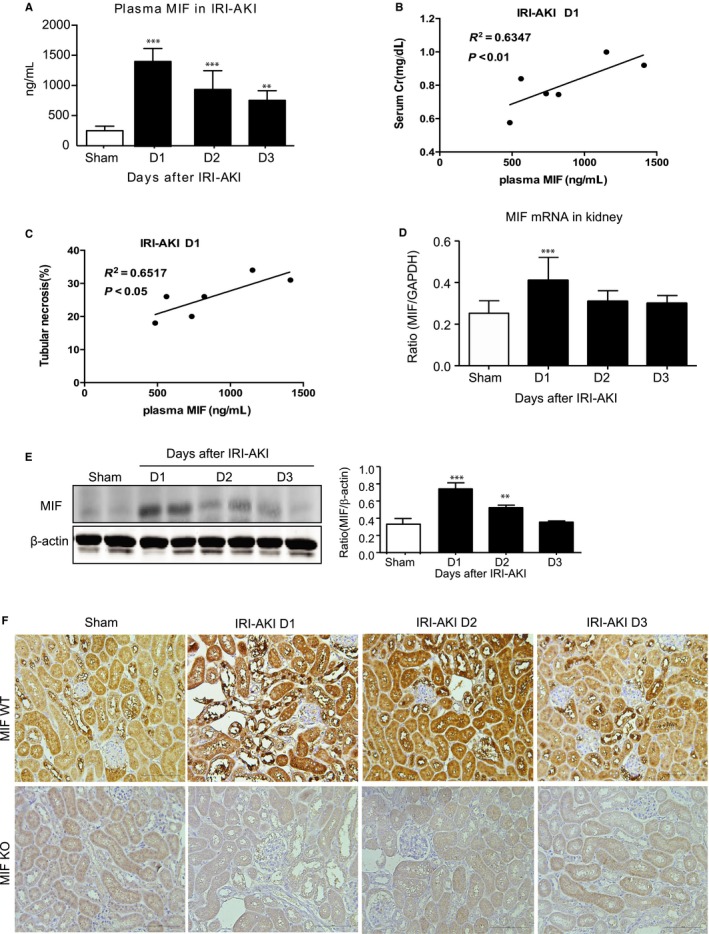
Plasma and renal MIF levels are significantly elevated in mice with IRI‐AKI. A, Plasma MIF levels in IRI‐induced AKI from day 1 to day 3. B, Linear correlation between plasma or urinary MIF levels and serum creatinine at day 1. C, Linear correlation between plasma levels of MIF and the degree of tubular necrosis at day 1. D, mRNA level of MIF in IRI‐induced AKI from day 1 to day 3. E, Immunohistochemistry reveals that renal MIF is predominantly produced by renal tubular epithelial cells in injured kidneys of mice with IRI‐AKI. Data are presented as the mean ± SEM. Each dot represents one AKI mouse. **P <* *0.01 and ***P <* *0.001 compared with the sham group (n = 6, per group). Scale bar: 100 μm

**Figure 3:**
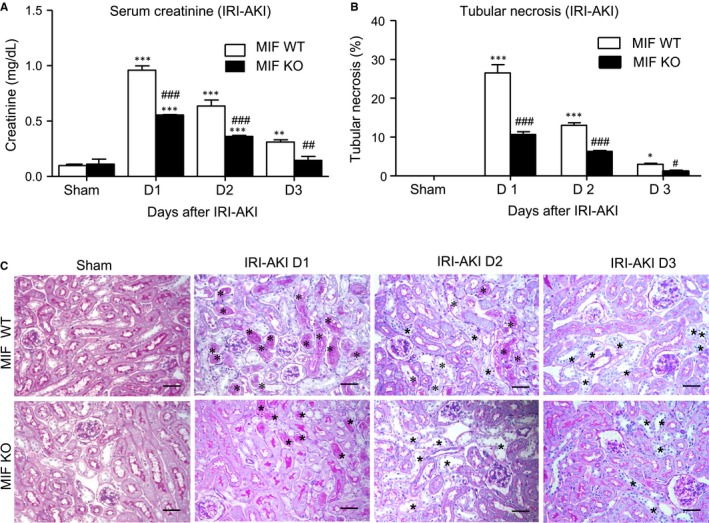
Deficiency of MIF protects against IRI‐AKI in mice. A, Serum creatinine levels in MIF wild‐type mice (MIF WT) and knockout (MIF KO) mice with IRI‐AKI were measured by ELISA. B, PAS staining and quantitative analysis of tubular necrosis in IRI‐ AKI from MIF WT and MIF KO mice. Note that mice lacking MIF are protected from AKI indicated by decreasing serum levels of creatinine and tubular necrosis (*). Results are presented as mean ± SEM (n = 6, per group). *P <* *0.05, **P <* *0.01 and **P <* *0.001 compared with the sham‐treated group; ^#^ *P *<* *0.05, ^##^ *P *<* *0.01 and ^###^ *P *<* *0.001 compared to MIF WT mice at the same time‐point. Scale bar: 50 μm

We further investigated whether there is a correlation between plasma MIF and IRI‐AKI in mice. In IRI‐AKI mice, the levels of plasma MIF rapidly increased as early as 6 hours, preceding the development of acute tubular necrosis and elevated serum creatinine at 24 hours after AKI (Figure S3). Plasma MIF remained high at 24 hours after AKI and was associated with a severe renal injury including elevated serum creatinine and severe acute tubular necrosis (Figure 2A‐C and Figure 3). Interestingly, plasma MIF was decreased gradually over the next 2 days of the recovery period from AKI (Figure 2A), which was associated with decline in serum creatinine and repair of acute tubular necrosis (Figure 3). Like the changes seen in plasma MIF, renal MIF mRNA and protein were also associated with the severity of AKI, peaking on day one after IRI‐AKI (Figure 2D‐E). Immunohistochemistry demonstrated that the renal tubular epithelial cells (TECs) was the dominant source of MIF production, particularly damaged TECs in the kidney in IRI‐AKI (Figure 2F).

### 3.3 MIF promotes IRI‐AKI in mice

We next examined whether MIF play a pathogenic role in IRI‐AKI in MIF WT/KO mice. As shown in Figure 3A, MIF WT mice showed a rapid increase in plasma MIF levels at the onset of AKI on day one with elevated serum creatinine, which was obviously reduced in after MIF deletion (Figure 3A). Strikingly, serum levels of creatinine decreased to nearly normal level in the MIF‐KO mice 72 hours after IRI‐AKI but not in WT mice. These findings suggest that a rapid increase in serum MIF contributes to the onset and severity of AKI and delays the recovery time after AKI. PAS staining revealed that deletion of MIF obviously reduced tubular necrosis in IRI‐AKI mice (Figure 3B and C).

### 3.4 MIF mediates IRI‐AKI by enhancing inflammation in the kidney

**Figure 4:**
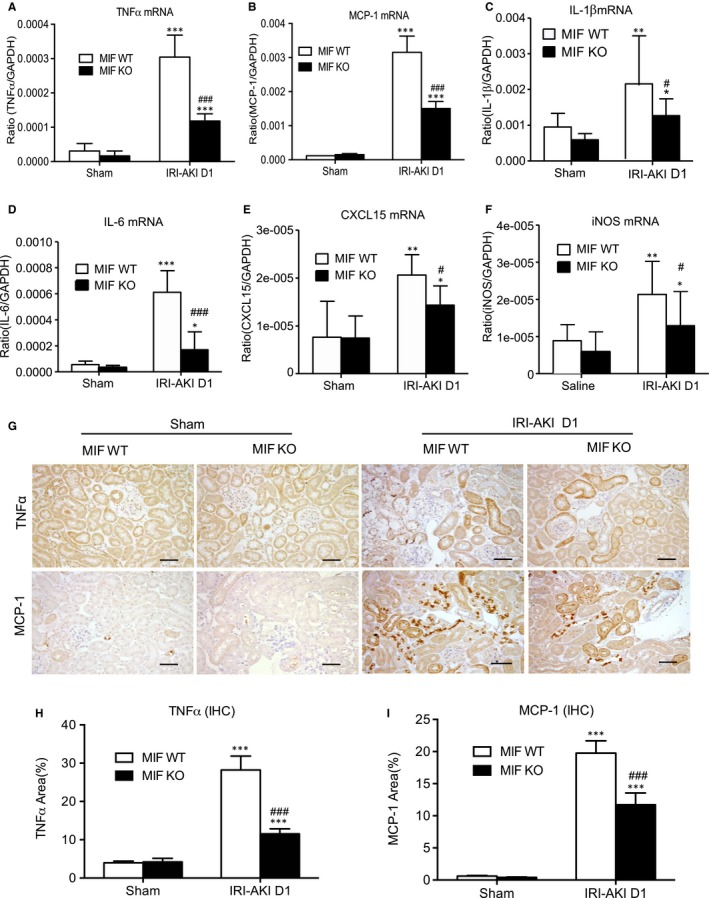
Real‐time PCR and immunohistochemistry show that deletion of MIF suppresses renal inflammatory cytokine expression in IRI‐AKI mice. A‐F, Renal cytokine mRNA expression of TNF‐α, MCP‐1, IL‐1β, IL‐6, CXCL15(IL‐8) and iNOS in IRI‐AKI mice. G‐I, Immunostaining and data analysis of TNF‐α and MCP‐1 in the kidneys of IRI‐AKI mice. Note that deletion of MIF largely suppresses the marked up‐regulation of TNF‐α, MCP‐1, IL‐1β, IL‐6, CXCL15(IL‐8) in the kidney suffered with IRI. Results are shown as mean ± SEM (n = 6, per group). *P <* *0.05, **P <* *0.01 and ***P <* *0.001 compared to the sham control; ^#^ *P *<* *0.05, ^##^ *P *<* *0.01 and ^###^ *P *<* *0.001 compared to MIF in WT mice at the same time‐point. Scale bar: 50 μm

**Figure 5:**
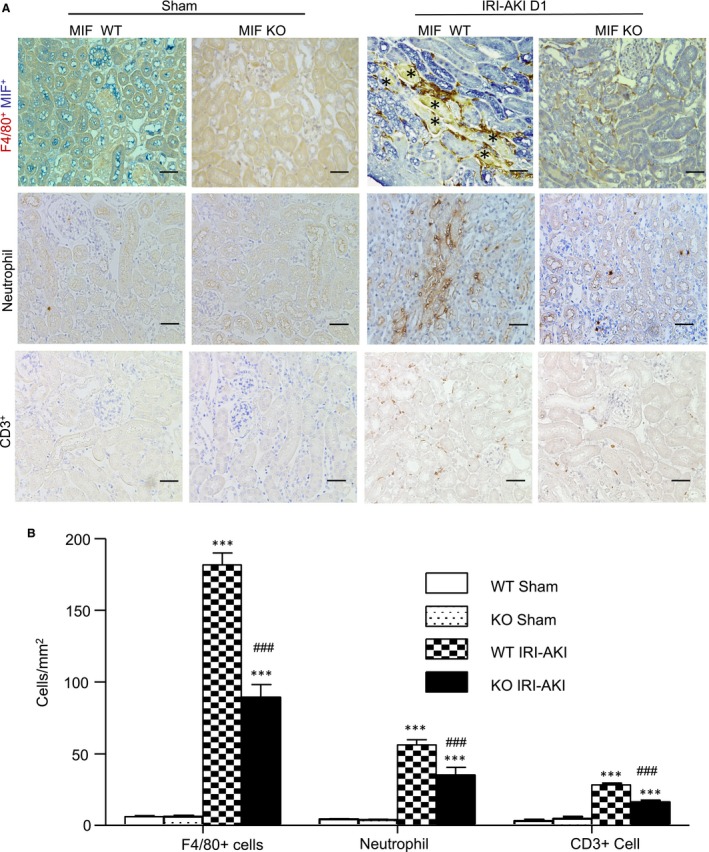
Deletion of MIF inhibits renal infiltration of F4/80 +  macrophages, neutrophils and CD3 +  T cells in IRI‐AKI mice. A, Immunostaining of F4/80 +  macrophages and MIF, neutrophils, and CD3 +  T cells in the kidneys of IRI‐AKI from MIF WT and MIF KO mice. Necrosis tubules (*) were clearly shown. B, Cognate quantitative analysis of F4/80 +  macrophages, neutrophils, and CD3 +  T cells. Note that a marked up‐regulation of tubular MIF (blue) is associated with the accumulation of F4/80 +  macrophages (brown). Results are presented as mean ± SEM (n = 6, per group). ***P <* *0.001 compared to the sham control; ^###^ *P *<* *0.001 compared to MIF WT mice at the same time‐point. Scale bar: 50 μm

Renal inflammation plays an important role in the process of AKI. We examined the mRNAs levels of encoding pro‐inflammatory cytokines and chemokines as MCP‐1, TNF‐α, IL‐6, IL‐1β, CXCL15 (also known as IL‐8 in human) and iNOS were greatly up‐regulated in the kidney of IRI‐AKI in MIF WT mice, which were markedly suppressed after MIF deletion (Figure 4A‐F). Immunohistochemistry also showed that TNF‐α and MCP‐1 were abundantly expressed in the kidney with an increase of F4/80^+^ macrophages, CD3^+^ T cells and neutrophils infiltration in the kidney of IRI‐AKI in MIF WT mice, these were largely suppressed after MIF deletion (Figure 4G‐I and Figure 5A and B). By two‐colour immunohistochemistry, we revealed that MIF expression was strongly associated with macrophage accumulation in the area of damaged tubules (Figure 5A), demonstrating that MIF maybe pathogenic in the development of AKI.

### 3.5 Expression of renal MIF is associated with up‐regulation of CD74 and TLR4 receptors in the kidney with AKI

**Figure 6:**
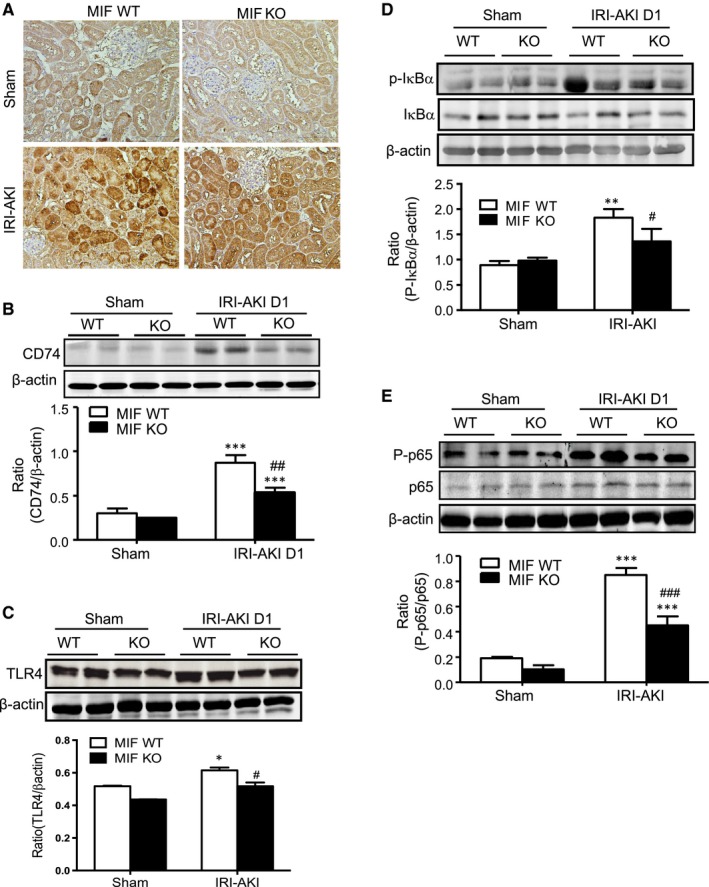
Western blot analysis shows that deletion of MIF inhibits renal CD74 and TLR4 expression, thus inhibits NF‐κB activation in IRI‐AKI kidney in mice. A, Immunostaining of the CD74 protein in the kidneys of IRI‐AKI mice at day 1. Scale bar: 100 μm. B, CD74 protein expression in the IRI‐AKI kidneys of MIF WT/KO mice at day 1. C, TLR4 expression in IRI‐AKI kidneys of MIF WT/KO mice. D, p‐IκBα and total IκBα in IRI‐AKI kidneys of MIF WT/KO mice. (E) Phosphorylation of NF‐κB/p65 in IRI‐AKI kidneys of MIF WT/KO mice. Results are presented as mean ± SEM (n = 6, per group). *P <* *0.05, **P <* *0.01 and ***P <* *0.001 compared to sham control. ^#^ *P *<* *0.05, ^##^ *P *<* *0.01 and ^###^ *P *<* *0.001 compared to MIF WT mice at the same time‐point

We then investigated the possible molecular mechanisms as to how MIF promotes AKI in mice. Immunohistochemistry showed that CD74, a MIF‐specific receptor, was highly expressed by both tubular epithelial cells and inflammatory cells infiltrating to the kidney of IRI‐AKI in mice, demonstrating that MIF‐CD74 signalling is highly active in the AKI kidney after IRI. All these changes were largely blocked after MIF deletion (Figure 6A). Similar results were also found by Western blot analyses demonstrating that a marked up‐regulation of renal CD74 in MIF WT mice was inhibited after MIF deletion (Figure 6B). NF‐κB signalling was tightly associated with MIF‐mediated inflammation and renal injury in IRI‐AKI. In contrast, deletion of MIF inactivated NF‐κB signalling by largely reducing the phosphorylation levels of NF‐κB/p65 and IKBα (Figure 6C and D). All these findings suggest that MIF may possibly act through CD74 to activate NF‐κB–dependent renal inflammation in AKI.

It has been reported that MIF is capable of inducing TLR4 to an induce inflammatory response in vitro,^25^ suggesting an alternative pathway of MIF/TLR4 interaction during the process of inflammation. As shown in Figure 6E, western blot analysis detected that TLR4 was markedly up‐regulated in the kidney after AKI, whereas, deletion of MIF down‐regulated TLR4 expression in the kidney.

## 4 DISSCUSSION

Macrophage migration inhibitory factor was a stress molecular that release quickly under disease conditions including surgery, sepsis, acute massive gastrointestinal bleeding or kidney diseases. It is reported that MIF is rapidly released and peaks within one hour during operation.^26^ Here, we reported that plasma MIF was closely correlated with the progression and regression in patients with AKI. Indeed, both circulating and urinary MIF was abundantly released at the initial onset of AKI and decreased to baseline levels when AKI recovered, which was independent of the causes of AKI. The elevated plasma and urinary MIF were also closely correlated with the serum levels of creatinine. This was consistent with previous studies in AKI patients with sepsis and liver transplantation in which high levels of plasma MIF are closely associated with poor clinical outcome,^14^, ^27^ whereas removal of circulating MIF by CRRT (renal replacement therapy) largely improved the AKI survival.^28^ Similarly, a close correlation between plasma MIF and serum creatinine was also found in the present study. Indeed, plasma MIF was rapidly released with the peak at 6 hours after AKI, preceding the elevated serum levels of creatinine and severe tubular necrosis at 24 hours after AKI, suggesting a causal effect of MIF in AKI. It is known that tubular epithelial cells are one of the major cell types for MIF production in the consequence of kidney injury.^24^, ^29^ In our study, we also found that MIF was highly expressed in injured cortical tubules as well as the MIF released to circulation by damaged tubular cells in response to IRI, this cause a marked increase of MIF levels in both plasma and urine. Similar results were reported in a mouse model of UUO.^16^ When AKI was resolved, renal MIF expression was reduced and MIF levels in plasma and urine declined to baseline again. These observations are consistent with the previous studies that levels of MIF reflect the severity of kidney injury in acute pyelonephritis,^13^ human renal allograft rejection,^30^ glomerulonephritis.^31^

The most significant thing is that we revealed MIF plays a pathogenic role in IRI‐AKI. This was supported by the finding that mice lacking MIF were protected against IRI‐induced AKI by significantly lowering serum creatinine and inhibiting TEC necrosis after IRI. This finding was consistent with our previous study in cisplatin‐induced AKI.^32^ Interestingly, inhibition of AKI by blocking MIF with a MIF inhibitor RPS19 reveals that targeting MIF may be a novel therapeutic potential for AKI.^32^ The pathogenic role of MIF has been reported in many inflammatory kidney diseases including rapidly progressive glomerulonephritis,^7^ lupus nephritis,^8^ anti‐glomerular basement membranous (GBM) glomerulonephritis,^9^ ANCA‐associated vasculitis,^10^ renal allograft rejection^11^, ^12^ and AKI.^13^ The present study showed that the development of AKI is associated with a rapid and amount release of MIF from injured tubular cells, which further trigger the inflammatory cytokines expression in the kidney as MCP‐1, TNF‐α, IL‐1β, IL‐6, CXCL15 (IL‐8 in human) and iNOS, these inflammation process further recruit large amount of macrophages, neutrophils and T cells to the injured kidney. Therefore, deletion of MIF protects IRI‐AKI by blunting these inflammatory responses. This was consistent with the pathogenic role of MIF in renal inflammatory diseases in which inhibition of MIF suppresses progressive renal injury in various forms of kidney diseases including anti‐GBM glomerulonephritis,^15^ septic shock,^28^ UUO^33^ and diabetic nephropathy.^34^ In contrast, a recent study reported that MIF is protective in AKI as high levels of plasma MIF in patients with cardiac surgery is associated with a reduced incidence of AKI and mice lacking MIF worsen AKI by inhibiting tubular epithelial cell proliferation in unilateral IRI‐induced AKI,^26^ which is also found in other two studies in which MIF or MIF‐2 inhibits AKI‐induced chronic renal injury by enhancing cell regeneration,^16^, ^35^ although serum levels of creatinine are lower in MIF KO mice at 24 hours after AKI^35^ which is consistent with our finding. The reason for this discrepancy remains largely unknown. It may be associated with the use of an unilateral IRI‐AKI mouse model in the Stoppe's study in which AKI is developed with minimal renal injury indicated by a marginal increase in serum creatinine, although serum MIF is not measured. In contrast, we used a more severe AKI mouse model by bilaterally clamping of both renal artery with more than threefold increase. It is highly possible that MIF at higher concentrations may cause inflammation as reported by many previous studies.^33^, ^36^, ^37^, ^38^ In addition, MIF may also promote cell proliferation when its concentration is not higher enough to cause inflammation.^35^, ^39^ Thus, MIF may have due role in renal inflammation and repair after AKI. This may explain enhanced tubular repair process in the unilateral IRI‐AKI mouse model as reported by Stoppe, whereas, much more severe renal injury occurred in our bilateral ischemic AKI with higher levels of MIF. The sex may be another factor associated with this discrepancy as male mice are more sensitive to the IRI‐induced AKI, whereas female mice seem more resistant.^40^, ^41^, ^42^ In Stoppe's study, female mice were used, in contrast, we used male mice in the present study. This may also explain the discrepancy between our study and Stoppe's study. Interestingly, the same group also reported that high level of MIF‐2 predicts organ dysfunction after myocardial ischemia/reperfusion injury.^43^ Nevertheless, the pathogenic role of MIF in AKI is warrant for further investigation.

It was possible that MIF may mediate AKI via CD74/TLR4‐NF‐κB signalling. It is well known that MIF acts via CD74 to exert its biological activities in many kidney diseases,^44^ and that deletion of CD74 can protect against NTS‐induced acute kidney disease.^45^ Thus, in the present study, genetic deletion of MIF led to the down‐regulation of CD74 and TLR4 and consequently to the inactivation of downstream NF‐κB signalling and the inhibition of NF‐κB‐dependent renal inflammation. Activated macrophages produce high levels of MIF,^46^ as well, this released MIF further enhances inflammation via recruiting and activating more macrophages. This may well explain macrophage‐mediated renal inflammation as a key process in the pathogenesis of AKI.^9^ MIF deficiency protected against IRI‐AKI by inhibiting CD74/TLR4‐NF‐κB‐dependent up‐regulation of MCP‐1, TNF‐α, IL‐1β, IL‐6, CXCL15 (IL‐8) and iNOS and by blocking the infiltration of neutrophils, macrophages and T cells to the injured kidney.

In conclusion, our study suggests that MIF may be pathogenic in AKI and levels of plasma and urinary MIF may correlate with the progression and regression of AKI. Targeting MIF may therefore serve a potential method for the therapy of IRI‐AKI.

## ACKNOWLEDGEMENTS

This study was supported by grants from the Science and Technology Project of Guangdong Province (Grant No. 408245702026), the Health and Medical Research Fund of Hong Kong (HMRF 14152321, 03140486) and the Research Grants Council of Hong Kong (GRF 14121816, 14117815, 14163317, and C7018‐16G).

## CONFLICTS OF INTEREST

The authors declare that there is no conflict of interest regarding the publication of this article.

## AUTHOR CONTRIBUTION

LJ and YT made an equal contribution on the performance of the experiments. HY and XHW provided the revised figures. XRH and PMK Tang provided the experimental guidance. JL, ZJH, ZJZ and QYH collected the clinical samples and performed ELISA measurements with XRH and PMK. JK, AM and APX provided the scientific guidance and GF‐R provided the MIF KO mice. ZHZ and HYL were the principal investigators for this project.

## Supporting information


** **

** **

** **

** **

** **

** **
