# Supplementary figures and images for: Macrophage migration inhibitory factor promotes renal injury induced by ischemic reperfusion
Source: J Cell Mol Med. 2019 Apr 9;23(6):3867–77. doi: 10.1111/jcmm.14234 (PMC6533527; doi:10.1111/jcmm.14234)

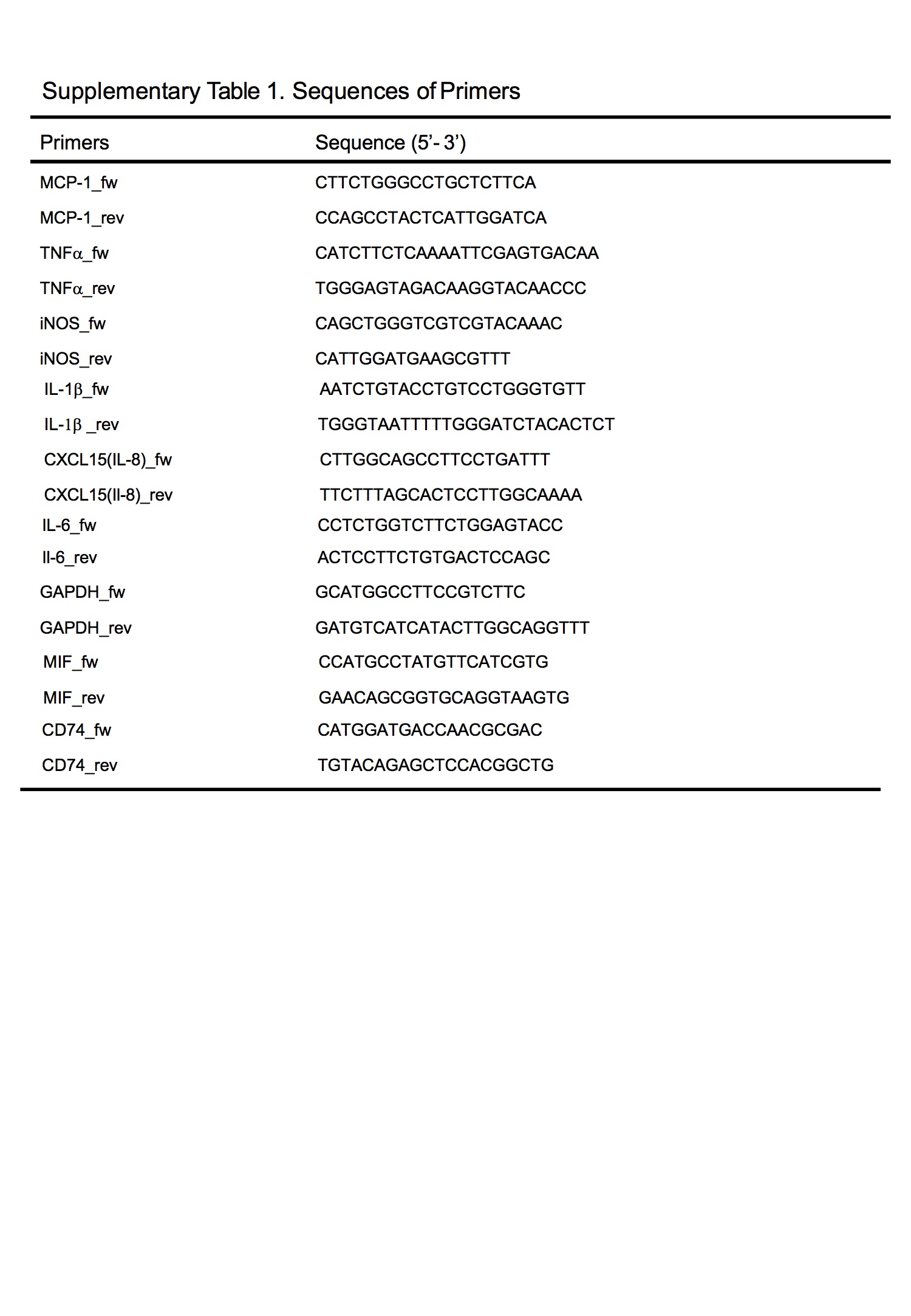

Supplement: Supplementary file 1 [file JCMM-23-3867-s001.jpg]

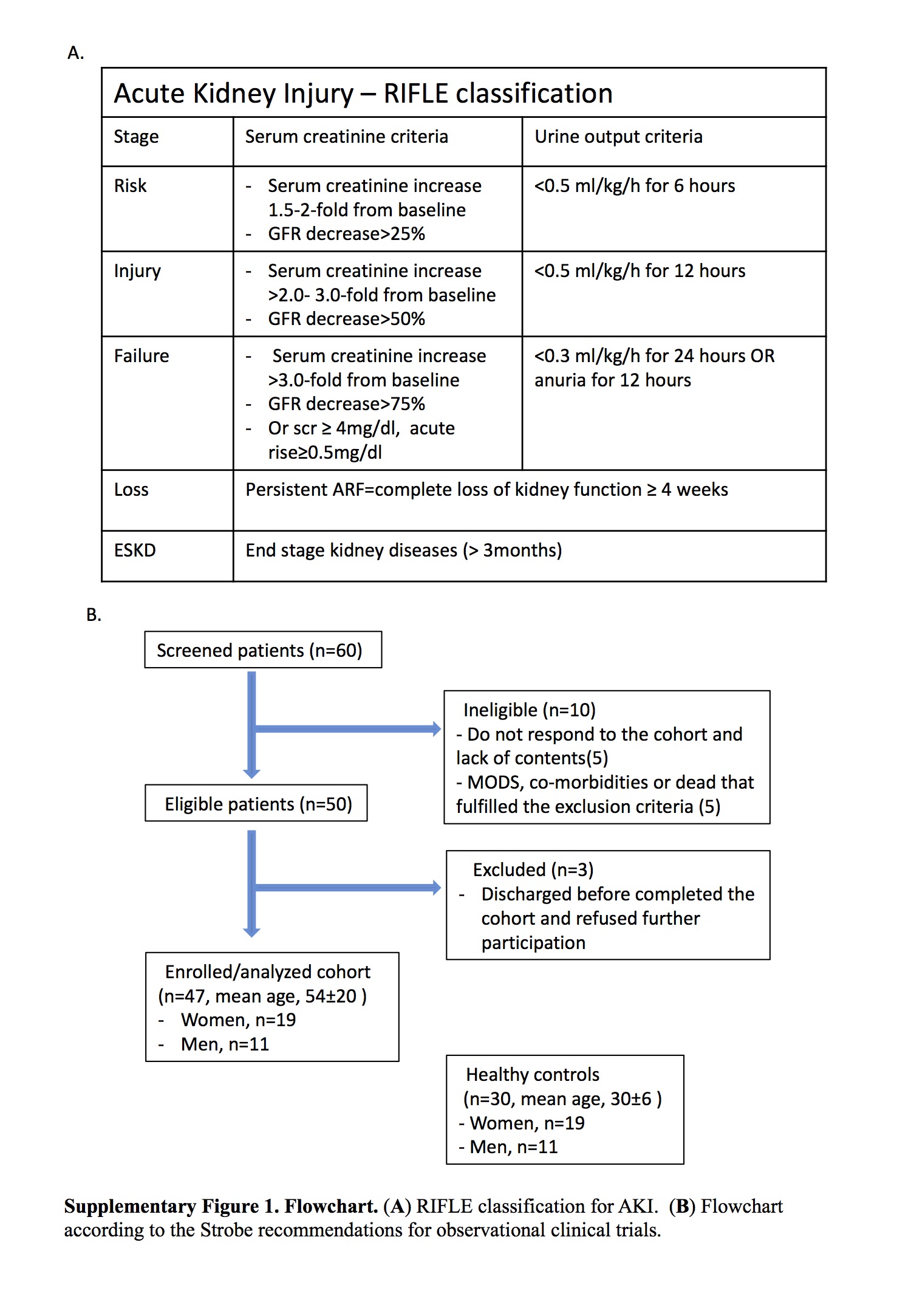

Supplement: Supplementary file 2 [file JCMM-23-3867-s002.jpg]

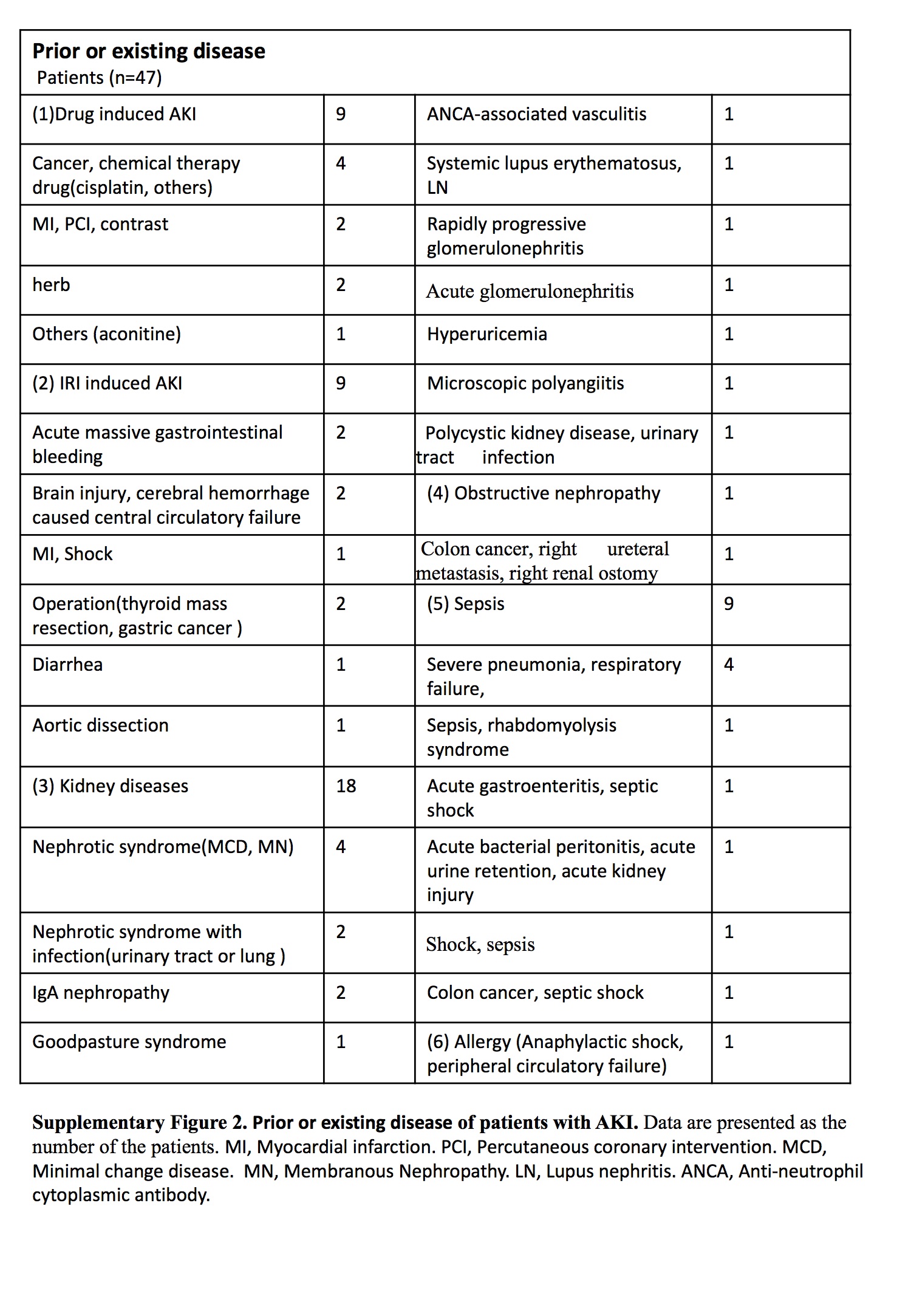

Supplement: Supplementary file 3 [file JCMM-23-3867-s003.jpg]

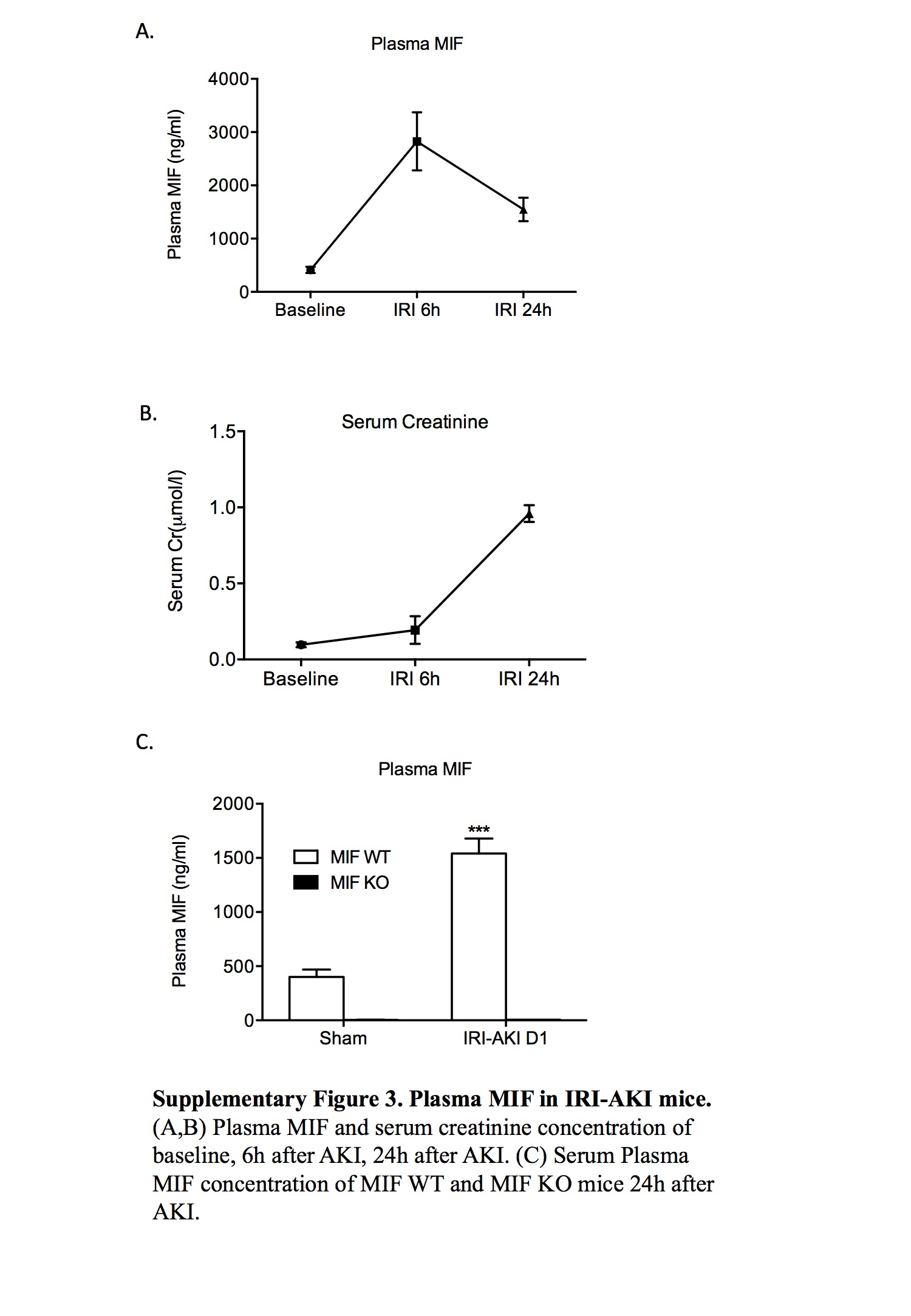

Supplement: Supplementary file 4 [file JCMM-23-3867-s004.jpg]

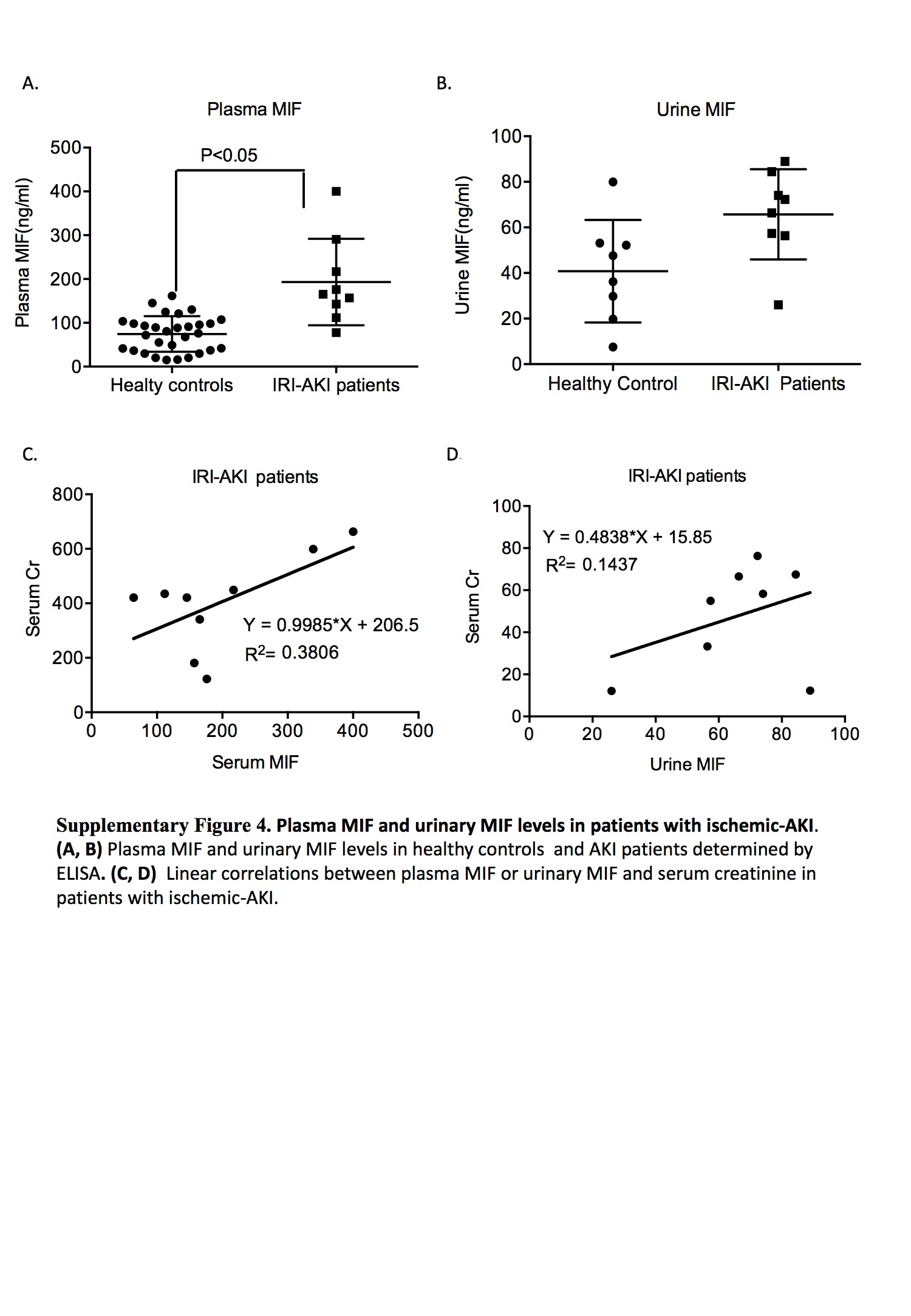

Supplement: Supplementary file 5 [file JCMM-23-3867-s005.jpg]
